# Imaging of carbonic anhydrase IX with an ^111^In-labeled dual-motif inhibitor

**DOI:** 10.18632/oncotarget.5254

**Published:** 2015-09-16

**Authors:** Xing Yang, Il Minn, Steven P. Rowe, Sangeeta Ray Banerjee, Michael A. Gorin, Mary Brummet, Hye Soo Lee, Soo Min Koo, Polina Sysa-Shah, Ronnie C. Mease, Sridhar Nimmagadda, Mohamad E. Allaf, Martin G. Pomper

**Affiliations:** ^1^ Russell H. Morgan Department of Radiology and Radiological Science, Johns Hopkins University School of Medicine, Baltimore, MD, USA; ^2^ The James Buchanan Brady Urological Institute and Department of Urology, Johns Hopkins University School of Medicine, Baltimore, MD, USA

**Keywords:** CAIX, single photon emission computed tomography, molecular imaging, renal cell carcinoma, indium-111

## Abstract

We developed a new scaffold for radionuclide-based imaging and therapy of clear cell renal cell carcinoma (ccRCC) targeting carbonic anhydrase IX (CAIX). Compound **XYIMSR-01**, a DOTA-conjugated, bivalent, low-molecular-weight ligand, has two moieties that target two separate sites on CAIX, imparting high affinity. We synthesized [^111^In]**XYIMSR-01** in 73.8–75.8% (n = 3) yield with specific radioactivities ranging from 118 – 1,021 GBq/μmol (3,200–27,600 Ci/mmol). Single photon emission computed tomography of [^111^In]**XYIMSR-01** in immunocompromised mice bearing CAIX-expressing SK-RC-52 tumors revealed radiotracer uptake in tumor as early as 1 h post-injection. Biodistribution studies demonstrated 26% injected dose per gram of radioactivity within tumor at 1 h. Tumor-to-blood, muscle and kidney ratios were 178.1 ± 145.4, 68.4 ± 29.0 and 1.7 ± 1.2, respectively, at 24 h post-injection. Retention of radioactivity was exclusively observed in tumors by 48 h, the latest time point evaluated. The dual targeting strategy to engage CAIX enabled specific detection of ccRCC in this xenograft model, with pharmacokinetics surpassing those of previously described radionuclide-based probes against CAIX.

## INTRODUCTION

Renal cell carcinoma (RCC) is the most common neoplasm of the kidney [1], with an estimated 60,000 patients diagnosed annually in the United States [2]. Among cases of RCC, the clear cell subtype (ccRCC) is the most prevalent, accounting for up to 70% of RCCs [3–5]. Common to ccRCC is loss of the Von Hippel-Lindau (*VHL*) tumor suppressor gene [6]. Loss of *VHL* in turn leads to over-expression of carbonic anhydrase IX (CAIX) [7], a membrane-associated enzyme responsible for catalyzing the reversible hydration of carbon dioxide to a bicarbonate anion and a proton [8, 9]. Over-expression of CAIX has been demonstrated in approximately 95% of ccRCC tumor specimens [10–12], making it a useful biomarker for this disease.

CAIX has limited expression in normal tissues and organs with the exception of the gastrointestinal tract, gallbladder and pancreatic ducts [8, 9, 13–15]. No report has demonstrated CAIX expression in normal renal parenchyma or benign renal masses [8, 9, 13–15]. Feasibility for the non-invasive detection of ccRCC based on CAIX expression has been proved with the radiolabeled antibody G250 [16] and its clinical potential has been reviewed [17]. However, antibodies as molecular imaging agents suffer from pharmacokinetic limitations, including slow blood and non-target tissue clearance (normally 2–5 days or longer) and non-specific organ uptake. Low-molecular-weight (LMW) agents demonstrate faster pharmacokinetics and higher specific signal within clinically convenient times after administration. They can also be synthesized in radiolabeled form more easily, and may offer a shorter path to regulatory approval [18–20].

Targeting CAIX with LMW inhibitors has proved challenging in part because fifteen human isoforms of carbonic anhydrase, with high sequence homology, have been identified. Those isoforms share common structural features, including a zinc-containing catalytic site, a central twisted β-sheet surrounded by helical connections, and additional β-strands. The isoforms, however, do vary widely in terms of intracellular location, expression levels, and tissue and organ distribution [8, 9]. Significant effort has been expended on development of sulfonamides and other LMW CAIX ligands for nuclear imaging of CAIX, but most reported agents have been fraught with low tumor uptake and significant off-target accumulation [21–26].

A new LMW CAIX targeting agent has recently been reported that is composed of two binding motifs, one accessing the CAIX active site and the other binding to an as yet unidentified site [27]. Conjugated with the infrared dye IRDye^®^750, the dual-motif inhibitor showed 10% ID/g tumor uptake. In comparison, agents targeting only the active site show 2% ID/g [27]. However, that optical agent also demonstrated high kidney as well as other non-specific organ uptake at 24 h post-administration. Additionally, utility of that agent for *in vivo* studies is somewhat limited due to the substantial attenuation of light emission through tissue inherent to optical agents. Such limitations call for an agent that retains affinity for CAIX, but clears rapidly from non-target tissues and can be detected with existing clinical instrumentation.

Here we report the synthesis and *in vivo* performance of [^111^In]**XYIMSR-01**, a modified dual-motif CAIX inhibitor with improved tumor uptake and pharmacokinetics for nuclear imaging of ccRCC. This reagent may enable imaging not only of metastatic ccRCC but also localized disease within the kidney due to relatively rapid clearance from normal renal tissue.

## RESULTS

Recently Wichert and co-workers [27] identified 4,4-bis(4-hydroxyphenyl)valeric acid/acetazolamide as a dual-motif CAIX inhibitor from a DNA-encoded chemical library [28–31]. The addition of a second binding motif significantly improved the potency of sulfonamide inhibitors (up to 40 times) [27], while also suggesting a solution to the problem of generating an isoform-selective CAIX inhibitor caused by conserved structures at the active site. We hypothesized that the slow renal clearance and high liver uptake of the reported optical agent might derive from the hydrophobicity of the molecule. To improve the pharmacokinetics, we replaced the IRDye^®^750 portion of the molecule with 1,4,7,10-tetraazacyclododecane-1,4,7,10-tetraacetic acid (DOTA), a more hydrophilic species that also enables convenient radiolabeling with metal isotopes for positron emission tomography (PET), single photon emission computed tomography (SPECT), and radiopharmaceutical therapy [32, 33]. We chose indium-111 as our initial radionuclide for its relatively long half-life (2.8 day) to enable extended monitoring of pharmacokinetics.

Chemical synthesis of **XYIMSR-01** was achieved as in Scheme 1. Following a reported procedure, key intermediate **1** was obtained *via* solid support synthetic methods [27]. We generated **XYIMSR-01** by conjugating the commercially available DOTA-NHS ester **7** with **1** in 82% yield. In(III) was incorporated into DOTA in nearly quantitative yield in 0.2 M NaOAc buffer at 60^°^C, providing the non-radiolabeled standard, [^113/115^In]**XYIMSR-01**. After optimization, baseline separation between **XYIMSR-01** and [^113/115^In]**XYIMSR-01** could be achieved by high performance liquid chromatography (HPLC).

**Figure:**
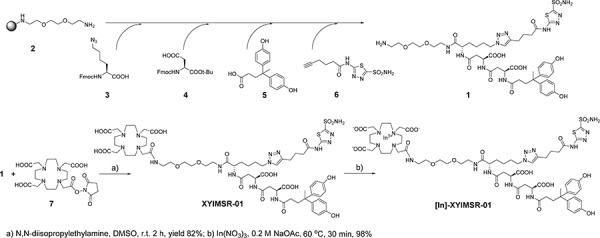


**Figure 1:**
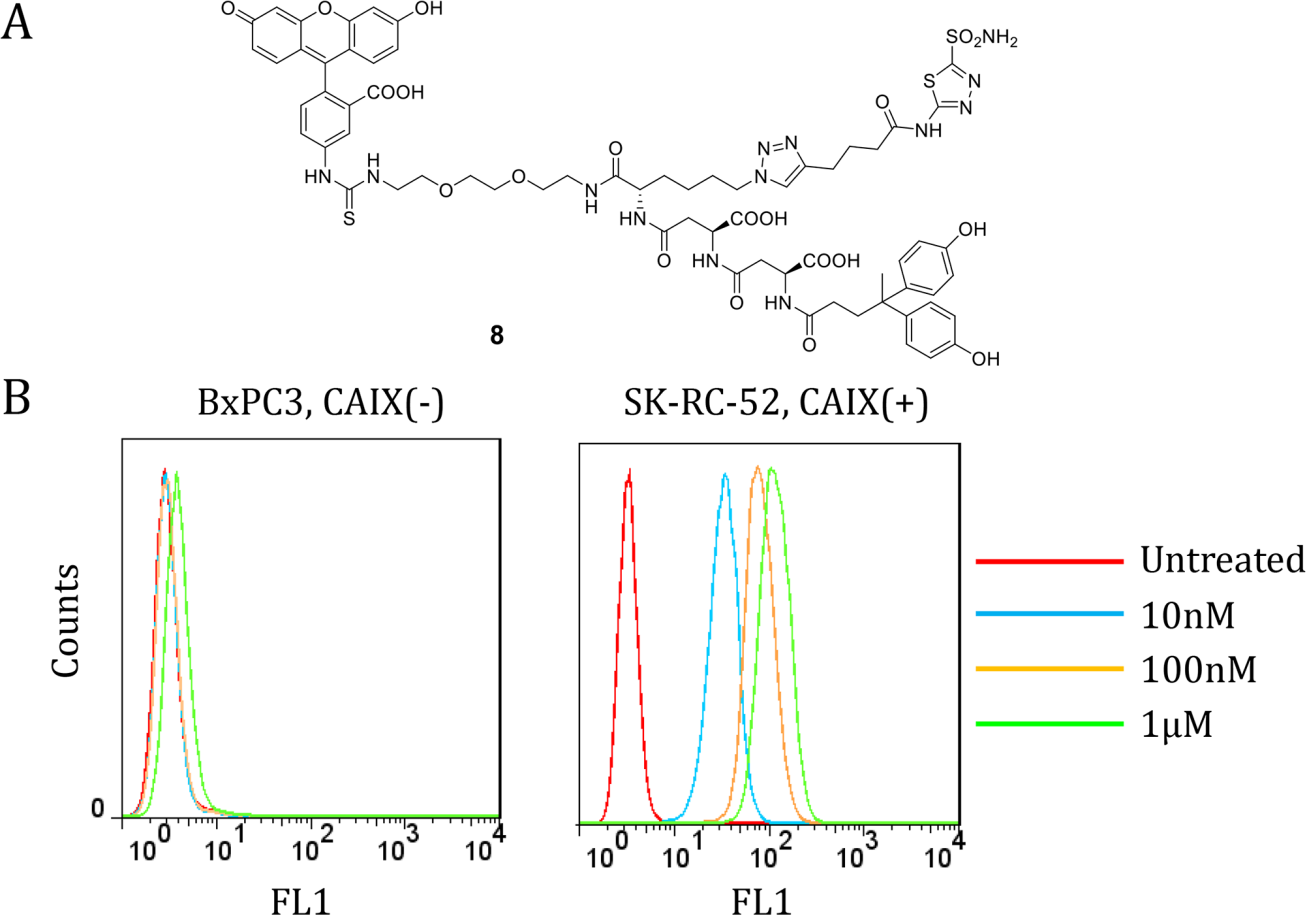
Dual-motif CAIX-targeting small molecule labeled with FITC 8 binds to CAIX-expressing SK-RC-52 cells **A.** Structure of **8**. **B.** FACS analysis of **8** for binding to CAIX-negative BxPC3 cells (left) and CAIX-expressing SK-RC-52 cells (right). Compound **8** showed significant binding to CAIX-expressing SK-RC-52 cells at concentrations as low as 10 nM.

**Figure 2:**
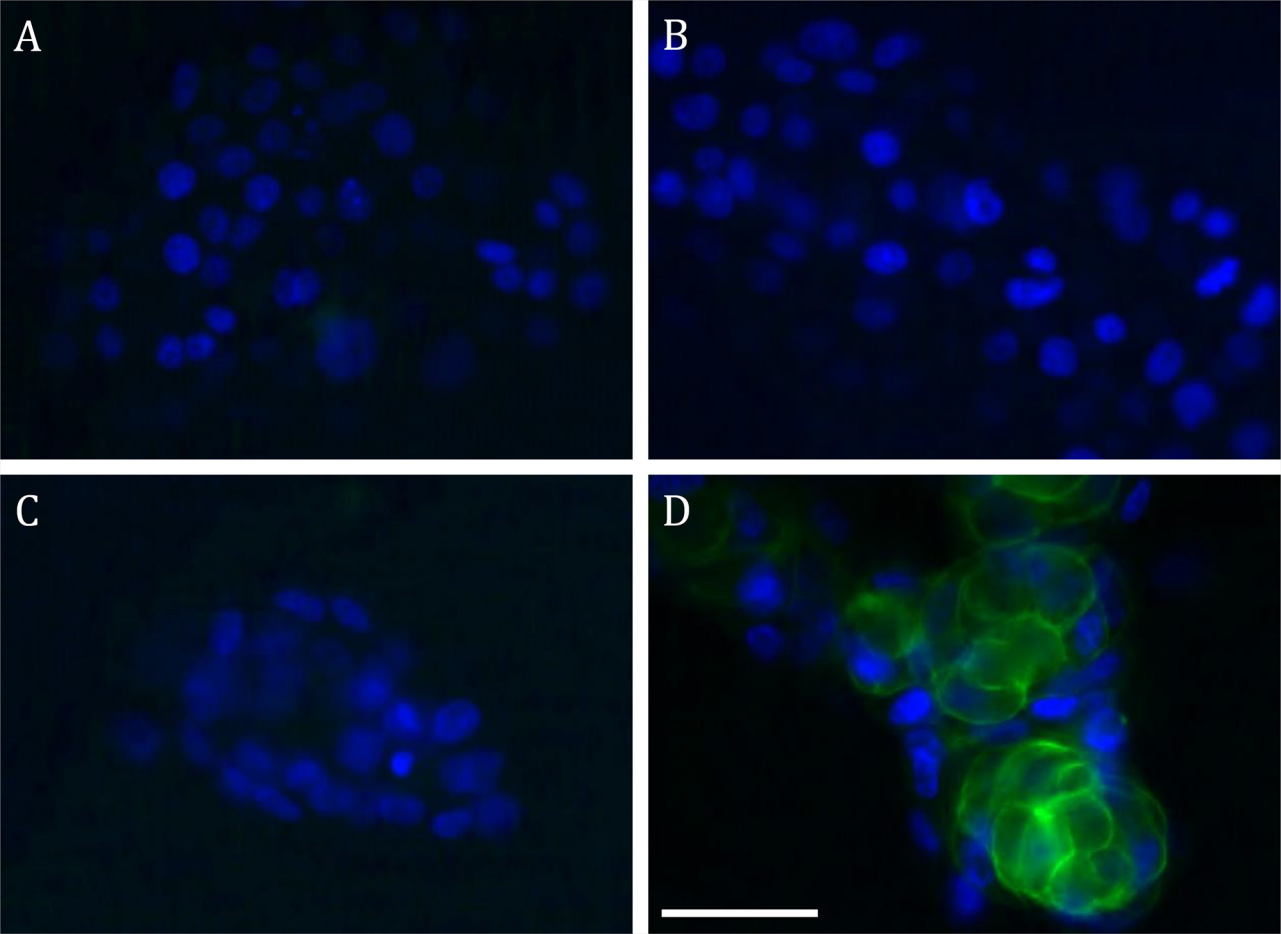
FITC-labeled 8 binds to the surface of CAIX-expressing SK-RC-52 cells Fluorescence microscopic analyses of BxPC3 **A** and **B,** and SK-RC-52 **C** and **D.** Cells are non-labeled (**A** and **C**) and labeled with **8** (**B** and **D**). Compound **8** bound to the cell surface of SK-RC-52. Scale bar = 50 μm.

**Figure 3:**
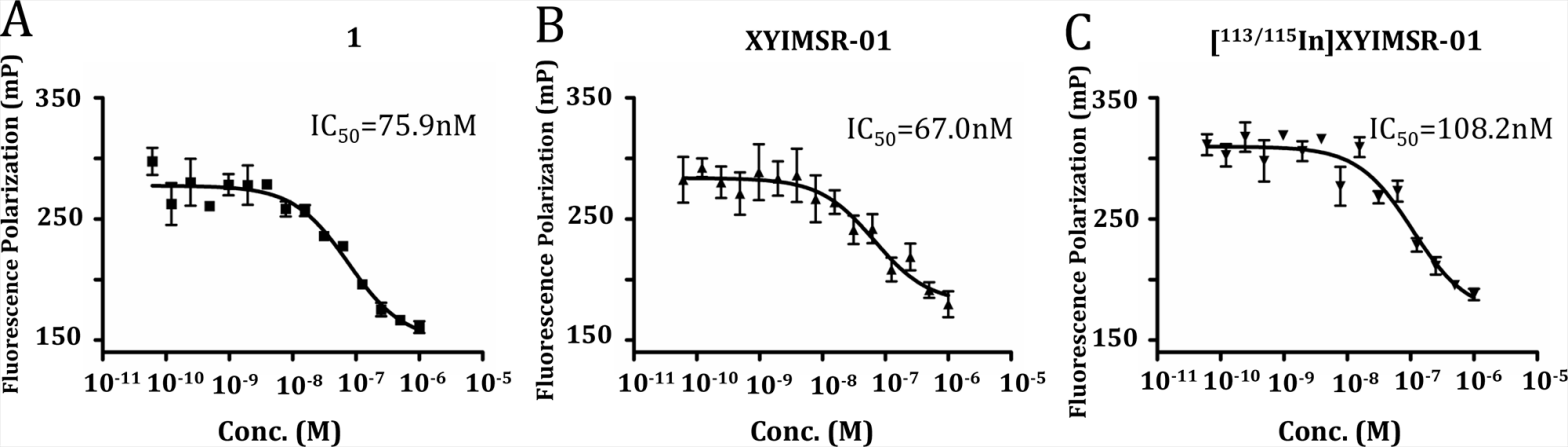
Compounds 1, XYIMSR-01 and [^113/115^In]XYIMSR-01 demonstrate high binding affinity to CAIX IC_50_ values of positive control CAIX targeting agent **1 A.** XYIMSR-01 **B.** and [^113/115^In]XYIMSR-01 **C.** were determined relative to the inhibition of fluorescence polarization of FITC labeled **8** with a known *K*_d_ of 0.2 nM for CAIX [27].

**Figure 4:**
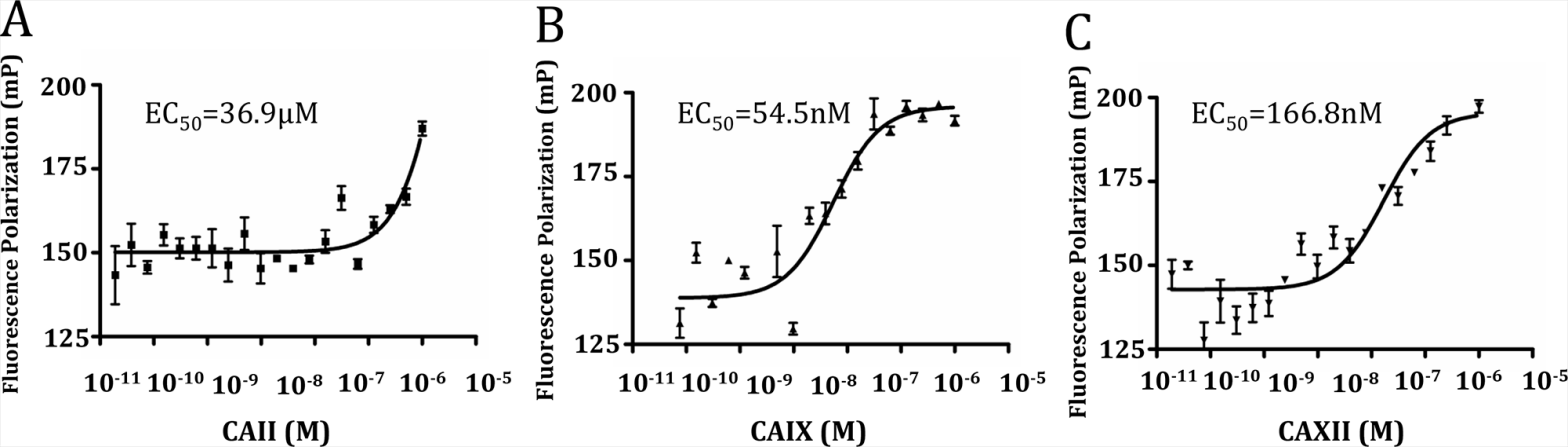
Compound 8 had higher binding affinity to CAIX compared to CAII and CAXII Saturation binding curves for CAII, CAIX, and CAXII were generated. 5 nM of compound **8** was titrated by increasing concentrations of CAII, CAIX, and CAXII and the resultant fluorescence polarization (mP) was measured, with EC_50_ values calculated.

We synthesized fluorescein isothiocyanate (FITC)-labeled **8** as a standard to measure CAIX binding affinities of the corresponding radiotracers. Compound **8** bound specifically to CAIX-expressing SK-RC-52 cells, but not to CAIX-negative BxPC3 cells measured by fluorescence activated cell sorting (Fig. 1A, B) [27]. CAIX-selective binding was confirmed by fluorescence microscopic analyses of SK-RC-53 and BxPC3 cells labeled with **8** (Fig. 2). Only SK-RC-52 cells were stained with **8** on the surface of the cells, where CAIX resides (Fig. 2D). In order to test the relative binding of **XYIMSR-01** and [^113/115^In]**XYIMSR-01** to CAIX we modified a competitive fluorescence polarization assay [34] for use with **8**. For the competitive binding assay, after optimization for background fluorescence, we chose concentrations of 80 nM and 100 nM for **8** and CAIX, respectively. As a positive control, we employed non-fluorescent **1**, which has a reported *K*_d_ value of 2.6 nM [27]. Increasing concentrations of **1**, **XYIMSR-01** and [^113/115^In]**XYIMSR-01** were incubated with CAIX for 30 min at room temperature. After **8** was added, fluorescence polarization signal was recorded. The IC_50_ values determined for **1**, **XYIMSR-01** and [^113/115^In]**XYIMSR-01** were 75.9, 67.0, and 108.2 nM, respectively (Fig. 3). These findings suggest that the DOTA-modified adducts were capable of binding CAIX with high affinity, on the order of positive control **1**. We took advantage of fluorescence polarization using **8** to measure relative binding affinities for other isoforms of the carbonic anhydrases. We chose to test one cytosolic (CAII) and an additional membrane-localized isoform (CAXII). Compound **8** exhibited poor binding affinity to cytosolic CAII (Fig. 4A) and about three-fold lower affinity to CAXII (Fig. 4C), indicating selectivity to CAIX.

**Figure 5:**
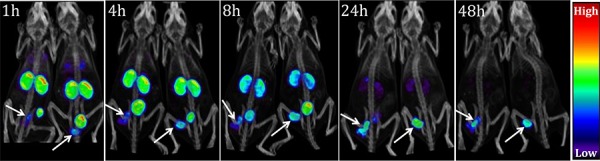
[^111^In]XYIMSR-01 enabled specific imaging of CAIX-expressing SK-RC-52 tumors SPECT/CT imaging of two mice harboring CAIX-expressing SKRC-52 tumors within the lower left flank. Images were obtained at 1, 4, 8, 24 and 48 h after injection of 14.8 MBq (400 μCi) of [^111^In]**XYIMSR-01** *via* the tail vein. Arrows indicate tumors.

We next investigated the capacity for [^111^In]**XYIMSR-01** to detect CAIX-expressing tumors *in vivo* using SPECT. The synthesis and purification of [^111^In]**XYIMSR-01** were achieved within 1.5 h in yields of 73.8–75.8% (n = 3) and with specific radioactivities of 118.4 – 1,021.2 GBq/μM (3,200 -27,600 Ci/mmol). [^111^In]**XYIMSR-01** was administered intravenously to two mice with SK-RC-52 flank tumors, followed by SPECT/CT. As shown in Fig. 5 and Supplementary Fig. 1, radiotracer uptake was observed within the tumors at 1 h post-injection. By 24 h post-injection, nearly all of the radioactivity in the kidneys and other organs had been eliminated, with tumor still retaining significant amounts of radiotracer. Image contrast improved even further by 48 h post-injection.

Biodistribution confirmed tumor-selective uptake and retention of [^111^In]**XYIMSR-01** observed in the imaging studies (Table 1 and Supplementary Table 1). At 1 h post-injection, 26.0% ID/g of radiotracer uptake was observed within the tumor. Tumor/blood and tumor/muscle ratios were 19.7 and 12.7, respectively. Major non-specific organ uptake was observed in kidney, lung, stomach, small intestine and liver (Table 1). Biodistribution studies conducted at later time points showed that radiotracer continued to clear from those organs while being retained within tumor. Considerable but lower affinity of [^111^In]**XYIMSR-01** to CAXII compared to CAIX (Fig. 4.) may explain the initial renal uptake of [^111^In]**XYIMSR-01** but more rapid early clearance from kidney than from tumor, since CAXII is abundant in kidney [35]. At 24 h post-injection, tumor/blood and tumor/muscle ratios reached 178 and 68, respectively. Importantly, tumor/kidney ratio reached 1.7, suggesting that it might be possible to detect local ccRCC in the kidney at 24 h. The enhanced hydrophilicity of [^111^In]**XYIMSR-01**, relative to the reported optical analog [27], may have contributed to the low liver uptake. The tumor/liver ratio for [^111^In]**XYIMSR-01** and the optical agent were 8.5 and 4.0 at 24 h, respectively [27]. All other organs showed tumor/organ ratios close to or higher than 10, indicating that suitable image contrast could be expected from these imaging agents. Biodistribution of [^111^In]**XYIMSR-01** simultaneously injected with non-radioactive competitor **1** showed competitive inhibition of uptake within tumors down to 1% ID/g at 24 h and 48 h post-injection, indicating CAIX-mediated binding (Table 1). The fast normal tissue clearance and the long-lasting tumor retention may enable applications to radiopharmaceutical therapy with appropriately selected therapeutic radiometals.

**Table 1:** Biodistribution of [^111^In]XYIMSR-01 at 1 h, 4 h, 8 h, 24 h and 48 h post-injection

Organs	1 h	4 h	8 h	24 h	24 h + Block	48 h	48 h + Block
Blood	1.34 ± 0.17	0.65 ± 0.06	0.48 ± 0.02	0.15 ± 0.02	0.03 ± 0.00	0.06 ± 0.02	0.03 ± 0.00
Heart	5.98 ± 0.53	2.91 ± 0.45	2.61 ± 0.35	1.16 ± 0.20	0.04 ± 0.01	0.84 ± 0.16	0.04 ± 0.01
Lung	45.85 ± 19.89	17.85 ± 3.55	17.39 ± 8.99	11.01 ± 3.71	0.12 ± 0.02	9.22 ± 1.25	0.09 ± 0.02
Pancreas	3.81 ± 0.72	1.54 ± 0.40	1.61 ± 0.28	0.69 ± 0.18	0.03 ± 0.00	0.59 ± 0.18	0.03 ± 0.01
Spleen	0.52 ± 0.04	0.51 ± 0.08	0.64 ± 0.07	0.69 ± 0.39	0.08 ± 0.01	0.67 ± 0.13	0.11 ± 0.03
Fat	1.03 ± 0.24	0.42 ± 0.22	0.45 ± 0.08	0.28 ± 0.19	0.02 ± 0.01	0.25 ± 0.08	0.03 ± 0.01
Brain	1.23 ± 1.10	0.45 ± 0.06	0.59 ± 0.09	0.71 ± 0.85	0.03 ± 0.00	0.41 ± 0.05	0.03 ± 0.01
Muscle (mm)	2.34 ± 2.19	1.01 ± 0.28	1.09 ± 0.15	0.35 ± 0.12	0.02 ± 0.00	0.39 ± 0.28	0.02 ± 0.00
Sm. intestine	9.37 ± 1.26	4.27 ± 0.69	4.31 ± 0.67	2.11 ± 0.33	0.08 ± 0.01	1.22 ± 0.44	0.08 ± 0.02
Liver	8.36 ± 0.73	4.00 ± 0.58	3.65 ± 0.65	3.02 ± 3.46	0.10 ± 0.02	1.65 ± 0.26	0.13 ± 0.04
Stomach	16.71 ± 2.46	7.91 ± 1.28	8.74 ± 1.26	3.31 ± 1.25	0.14 ± 0.02	1.82 ± 0.43	0.14 ± 0.03
Kidney (kid)	71.26 ± 8.74	41.52 ± 6.07	28.79 ± 21.35	15.29 ± 1.69	0.68 ± 0.14	8.78 ± 1.89	0.45 ± 0.09
Bladder	4.90 ± 4.96	2.68 ± 1.89	2.28 ± 0.51	0.74 ± 0.17	0.20 ± 0.07	0.38 ± 0.18	0.14 ± 0.03
Tumor	26.01 ± 5.74	20.83 ± 6.25	34.00 ± 15.16	25.62 ± 17.67	1.41 ± 0.20	13.92 ± 6.67	1.22 ± 0.54
Tumor/blood	19.7 ± 4.8	31.9 ± 9.4	77.0 ± 32.5	178.1 ± 145.4	45.2 ± 9.7	212.0 ± 41.4	45.4 ± 13.8
Tumor/mm	12.7 ± 5.8	21.4 ± 7.2	34.2 ± 16.0	68.4 ± 29.0	91.4 ± 11.1	52.0 ± 21.0	75.1 ± 17.7
Tumor/kid	0.36 ± 0.06	0.50 ± 0.15	3.1 ± 3.1	1.7 ± 1.2	2.1 ± 0.3	1.5 ± 0.5	2.7 ± 0.8

Results are expressed as the percentage injected dose per gram (%ID/g) of tissue, *n* = 5.

Despite intensive effort expended in the development of CAIX inhibitors designed to engage only the active site, nuclear imaging analogs have continued to demonstrate limited success, showing < 2% ID/g within tumor and high radiotracer uptake within kidney and liver [21–26]. Peptides that bind to the surface of CAIX may provide an alternative solution to selective targeting, but they are limited by low potency and *in vivo* stability [27]. Dual-motif ligands that may concurrently engage the CAIX active site and surface binding demonstrated high potency and tumor uptake for [^111^In]**XYIMSR-01** and for the previously reported optical agent [27]. The hydrophilicity of [^111^In]**XYIMSR-01**, with multiple carboxylates and heteroatoms, improved non-target organ clearance, including that from kidney and liver. Further detailed studies on the selectivity of [^111^In]**XYIMSR-01** for CAIX and its stability *in vivo* are under way.

## METERIALS AND METHODS

### Chemistry

Solvents and chemicals obtained from commercial sources were of analytical grade or better and used without further purification. Fmoc-protected azidolysine, HBTU, and N-α-fmoc-L-aspartic acid α-tert-butyl ester were purchased from Chem Impex International, Inc. (Wooddale, IL). Carrier-free [^111^In]InCl_3_ was purchased from MDS Nordion (Ottawa, ON, Canada). DOTA-NHS-ester (1,4,7,10-tetraazacyclododecane-1,4,7,10-tetraacetic acid mono N-hydroxysuccinimide ester) was purchased from Macrocyclics, Inc. (Dallas, TX). Indium (III) nitrate, triethylsilane (Et_3_SiH), N,N-diisopropylethylamine (DIEA), triethylamine (TEA), piperidine, 4,4-bis(4-hydroxyphenyl)valeric acid, copper iodide (CuI), and tris[(1-benzyl-1H-1,2,3-triazol-4-yl) methyl] amine (TBTA) were purchased from Sigma-Aldrich (Saint Louis, MO). Pre-loaded O-bis-(aminoethyl)ethylene glycol on trityl resin was purchased from EMD Millipore (Billerica, MA). Flash chromatography was performed using MP SiliTech 32–63 D 60Å silica gel purchased from Bodman (Aston, PA). Recombinant human CAIX was purchased from R&D Systems (Minnepolis, MN). ^1^H NMR spectra were recorded on a Bruker Ultrashield 500 MHz spectrometer. Chemical shifts (δ) were reported in ppm downfield by reference to proton resonances resulting from incomplete deuteration of the NMR solvent. ESI mass spectra were obtained on a Bruker Daltonics Esquire 3000 Plus spectrometer (Billerica, MA).

HPLC purification of non-labeled compounds was performed using a Phenomenex C18 Luna 10 × 250 mm^2^ column on an Agilent 1260 infinity LC system (Santa Clara, CA). HPLC purification of radiolabeled (^111^In) ligand was performed on another Phenomenex C18 Luna 10 × 250 mm^2^ and a Varian Prostar System (Palo Alto, CA), equipped with a Varian ProStar 325 UV-Vis variable wavelength detector and a Bioscan (Poway, CA) Flow-count in-line radioactivity detector, all controlled by Galaxie software. The specific radioactivity was calculated as the ratio of the radioactivity eluting at the retention time of product during the preparative HPLC purification to the mass corresponding to the area under the curve of the UV absorption. The purity of tested compounds as determined by analytical HPLC with absorbance at 254 nm was > 95%.

### 2,2′,2′'-(10-((14S,18S,22S)-18,22-dicarboxy-27,27-bis(4-hydroxyphenyl)-2,13,16,20,24-pentaoxo-14-(4-(4-(4-oxo-4-((5-sulfamoyl-1,3,4-thiadiazol-2-yl)amino)butyl)-1H-1,2,3-triazol-1-yl)butyl)-6,9-dioxa-3,12,15,19,23-pentaazaoctacosyl)-1,4,7,10-tetraazacyclododecane-1,4,7-triyl)triacetic acid (XYIMSR-01)

N^4^-((S)-1-((2-(2-(2-aminoethoxy)ethoxy)ethyl)amino)-1-oxo-6-(4-(4-oxo-4-((5-sulfamoyl-1,3,4-thiadiazol-2-yl)amino)butyl)-1H-1,2,3-triazol-1-yl)hexan-2-yl)-N^2^-((S)-3-(4,4-bis(4-hydroxyphenyl)pentanamido)-3-carboxypropanoyl)-L-asparagine (**1**) 19 mg (0.017 mmol), DOTA-NHS **7** 16 mg (0.021 mmol) and N,N-diisopropylethylamine 150 μL were mixed in 2 mL DMSO. The reaction was stirred at room temperature for 2 h. Solvent was removed under vacuum. 21 mg (0.014 mmol) of product **XYIMSR-01** was obtained as a white powder after purification by HPLC in 82% yield. HPLC conditions: Phenomenex, Luna 10 × 250 mm, 10 μ. Gradient 10/90/0.1 to 50/50/0.1 MeCN/H_2_O/TFA, 0–10 min, flow 10 mL/min. Product eluted at 6.3 min.

^1^H-NMR (500 MHz, DMSO-d6): δ 13.01 (s, 1H), 12.77 (br. 2H), 9.17 (br. s, 2H), 8.53 (br, 1H), 8.33 (s, 2H), 8.19 (d, J = 8.0, 1H), 8.09 (d, J =7.9, 1H), 7.91 (d, J = 8.1, 1H), 7.88 (t, J = 6.0, 1H), 7.84 (s, 1H), 7.45 (br. 2H), 6.92 (d, J = 8.4, 4H), 6.64 (d, J =8.4, 4H), 4.54 – 4.44 (m, 2H), 4.24 (t, J = 7.2, 2H), 4.17 (td, J = 8.3, 5.5, 1H), 4.0–3.0 (36H, overlap with water signal), 2.65 (t, J = 7.5, 2H), 2.64 – 2.55 (m, 4H), 2.51 – 2.41 (m, 2H), 2.17 (t, J = 8.2, 2H) 1.94 (m, J = 7.5, 2H), 1.88 – 1.82 (m, 2H), 1.75 (m, J = 7.5, 2H), 1.66 – 1.60 (m, 1H), 1.53 – 1.46 (m, 1H), 1.45 (s, 3H), 1.28 – 1.17 (m, 2H). MS, calcd. for C_61_H_88_N_16_NaO_22_S_2_^+^ [M+Na]^+^: 1483.6; Found: 1483.4.

### ^113/115^Indium(III) 2,2′,2′'-(10-((14S,18S,22S)-18,22-dicarboxy-27,27-bis(4-hydroxyphenyl)-2,13,16,20,24-pentaoxo-14-(4-(4-(4-oxo-4-((5-sulfamoyl-1,3,4-thiadiazol-2-yl)amino)butyl)-1H-1,2,3-triazol-1-yl)butyl)-6,9-dioxa-3,12,15,19,23-pentaazaoctacosyl)-1,4,7,10-tetraazacyclododecane-1,4,7-triyl)triacetate ( [^113/115^In] XYIMSR-01)

2 mg (0.0013 mmol) **XYIMSR-01** was dissolved in 1 mL of 0.2 M NaOAc water solution. Then, 20 μL of a solution of In(NO_3_)_3_ containing 0.6 mg of In(NO_3_)_3_ was added. The solution was kept at 60°C for 30 min. 2.0 mg [^113/115^In]**XYIMSR-01** was obtained as white crystals after purification by HPLC in 98% yield. HPLC conditions: Phenomenex, Luna 10 × 250 mm, 10 μ. 20/80/TFA MeCN/H_2_O /TFA, flow 10 mL/min. Product eluted at 10.6 min.

MS, calcd. for C_61_H_85_InN_16_NaO_22_S_2_^+^ [M+Na]^+^: 1595.4; Found: 1595.3.

### Radiosynthesis of [^111^In]XYIMSR-01

20 μg **XYIMSR-01** was dissolved in10 μL of 0.2M NaOAc followed by addition of 3.3 mCi of ^111^InCl_3_ solution to provide a final pH = 5.5–6. The mixture was heated in a water bath at 65°C for 30 min. Radiolabeling was monitored by HPLC. At completion, the reaction mixture was diluted with 1 mL of water then loaded onto a preparative HPLC column for purification. Retention times for the radiolabeled compound, [^111^In]**XYIMSR-01**,and starting material, **XYIMSR-01**,were optimized to the point of baseline separation, with [^111^In]**XYIMSR-01** eluting first. 2.5 mCi of [^111^In]**XYIMSR-01** was obtained at a radiochemical yield of 75.8% in specific radioactivites of 118.4 GBq/μmol (3,200 Ci/mmol). The identity of the radiolabeled product was confirmed by co-injection with [^113/115^In]**XYIMSR-01** and co-elution on HPLC with the same condition. Another two syntheses were performed under similar conditions. The average yield was 74% (n = 3). The specific activities ranged from 118 to 1021.2 GBq/μmol (3,200 to 27,600 Ci/mmol). For preparative runs, the HPLC solvent was removed under vacuum. [^111^In]**XYIMSR-01** was formulated in phosphate-buffered saline (PBS) for the imaging study. HPLC conditions: Phenomenex, Luna 10 × 250 mm, 10μ. 19/81/0.1 MeCN/H_2_O/TFA, flow 4 mL/min. Product eluted at 28.3 min, while starting material eluted at 30.2 min.

### Cell lines and mouse models

Animal experiments were performed in accordance with protocols approved by the Johns Hopkins Animal Care and Use Committee (ACUC). Six-week-old female NOD/SCID mice were purchased from the Animal Resource Core of the Sidney Kimmel Comprehensive Cancer Center of Johns Hopkins and were subcutaneously injected in the lower left flank with 1 × 10^6^ SK-RC-52 cells in RPMI 1640 GlutaMAX™ media (Life Technologies, Frederick, MD) supplemented with 1% fetal bovine serum (FBS). Mice were monitored for tumor size and used for SPECT/CT imaging when the size of the tumor reached 100 mm^3^.

### FACS analysis

CAIX-positive SK-RC-52 and CAIX-negative BxPC3 cells were maintained in RPMI 1640 media supplemented with 10% FBS and 1 × penicillin-streptomycin in a 37°C humidified incubator. Cells were detached from the flask with trypsin and reconstituted in RPMI 1640 media supplemented with 1% FBS at a density of 1 × 10^6^ cells per mL. FITC-labeled **8** was added to the cells at the indicated concentration and incubated at room temperature for 30 min. Cells were washed twice with the same media for staining and analyzed using the FACSCalibur (BD Bioscience, San Jose, CA) instrument.

### Microscopic analyses

CAIX-positive SK-RC-52 and CAIX-negative BxPC3 cells were seeded on to 8-well chamber glass slides (Lab-Tek^®^IICC^2^™, Nunc, Rochester NY) and incubated in RPMI 1640 media supplemented with 10% FBS and 1 × penicillin-streptomycin in a 37°C humidified incubator for 48 h. Cells were stained with 100 nM of FITC-labeled **8** for 1 h in the same growth media followed by washing twice with the same media. Cells were fixed with 10% formaldehyde (Sigma-Aldrich, Saint Louis, MO) and washed three times with PBS. Cells were treated with 20 nM DAPI (4′,6-diamidino-2-phenylindole) in PBS. The chambers were removed and the Vectashield mounting solution (Vector Laboratories, Inc., Burlingame, CA) was added to the sample. Fluorescence microscopic images were taken using the Nikon Eclipse 80i epifluorescence microscope (Nikon Instruments Inc., Melville, NY) and the images were processed by the Element software (Nikon Instruments Inc.).

### Competitive fluorescence polarization assay [34]

Fluorescence polarization (FP) experiments were performed in 21 μL of the assay buffer (12.5 mM Tris-HCl, pH 7.5, 75 mM NaCl) in black flat bottom 384-well microplates (Corning, Inc., New York, NY). The FP reaction employed 100 nM of purified CAIX (R&D systems, Minneapolis, MN) and 80 nM FITC-labeled **8** [27] within the assay buffer. The FP values were measured as mP units using the Victor3 multi-label plate reader equipped with excitation (485 nm) and emission (535 nm) filters (Perkin Elmer, Waltham, MA). 100 nM CAIX was incubated with serially diluted (from 1 μM to 61 fM) concentrations of the three targeting molecules, **1**, **XYIMSR-01**, and [^113/115^In]**XYIMSR-01** for 30 min at room temperature in 384-well plates. 80 nM **8** was added to each well and the reaction was incubated for 30 min at room temperature followed by FP measurement. Experiments were carried out in triplicate and the concentration resulting in 50% response (IC_50_) was calculated in GraphPad Prism 5 (GraphPad Software, La Jolla, CA) using the sigmoidal dose-response regression function.

### Fluorescence polarization for affinity comparison

Human recombinant CAII, CAIX, and CAXII were purchased from R&D systems (Minneapolis, MN). 5 nM of FITC-labeled **8** were mixed with serially diluted (from 1 μM to 19 fM) isoforms of the carbonic anhydrases in PBS within 384 well Small Volume™ LoBase Microplates (Greiner Bio-One, Frickenhausen Germany). The mixtures were incubated at room temperature for 1 h. Fluorescence polarization was measured using a Safire2™ plate reader (Tecan, Morrisville, NC), with 475 nm excitation and 530 nm emission wavelengths.

### Imaging

Mice harboring subcutaneous SK-RC-52 tumors with the lower left flank were injected with 14.8 MBq (400 μCi) of [^111^In]**XYIMSR-01** in 250 μL of PBS (pH = 7.0) intravenously (tail vein). Anesthesia was then induced with 3% isofluorane and maintained at 2% isoflurane. Physiologic temperature was maintained with an external light source while the mouse was on the gantry. Imaging employed a CT-equipped Gamma Medica-Ideas SPECT scanner (Northridge, CA). SPECT data were acquired in 64 projections at 65 s per projection using medium energy pinhole collimators. A CT scan was performed in 512 projections at the end of each SPECT scan for anatomic co-registration. CT and SPECT scans were performed at 1, 4, 8, 24, and 48 h post-injection of [^111^In]**XYIMSR-01**. Imaging data sets were reconstructed using the manufacturer's software. Display of images utilized Amide software (Dice Holdings, Inc. NY).

### Biodistribution

Mice bearing SK-RC-52 xenografts within the lower left flank were injected intravenously with 740 kBq (20 μCi) of [^111^In]**XYIMSR-01** in 200 μL of PBS. For *in vivo* competition (binding specificity) studies, tumor-bearing mice were injected with 740 kBq (20 μCi) of [^111^In]**XYIMSR-01** and 200 nmole of **1** in 200 μL of PBS concurrently. At 1 h, 4 h, 8 h, 24 h and 48 h post-injection, mice were sacrificed by cervical dislocation and the blood was immediately collected by cardiac puncture. Heart, lungs, pancreas, spleen, fat, brain, muscle, small intestines, liver, stomach, kidney, urinary bladder, and tumor were collected. Each organ was weighed and the tissue radioactivity was measured with an automated gamma counter (1282 Compugamma CS, Pharmacia/LKBNuclear, Inc., Mt. Waverly, Vic. Australia). The percentage of injected dose per gram of tissue (% ID/g) was calculated by comparison with samples of a standard dilution of the initial dose. All measurements were corrected for radioactive decay.

Data were expressed as mean ± standard deviation (SD). Prism software (GraphPAD, San Diego, California) was used to determine statistical significance. Statistical significance was calculated using a paired t test. *P*-values < 0.0001 were considered significant.

## SUPPLEMENTARY FIGURE AND TABLE


