# Supplementary material for: Imaging of carbonic anhydrase IX with an 111In-labeled dual-motif inhibitor
Source: Oncotarget. 2015 Sep 16;6(32):33733–42. doi: 10.18632/oncotarget.5254 (PMC4741798; doi:10.18632/oncotarget.5254)
Supplement: Supplementary file 1 [file oncotarget-06-33733-s001.pdf]

SUPPLEMENTARY FIGURE AND TABLE

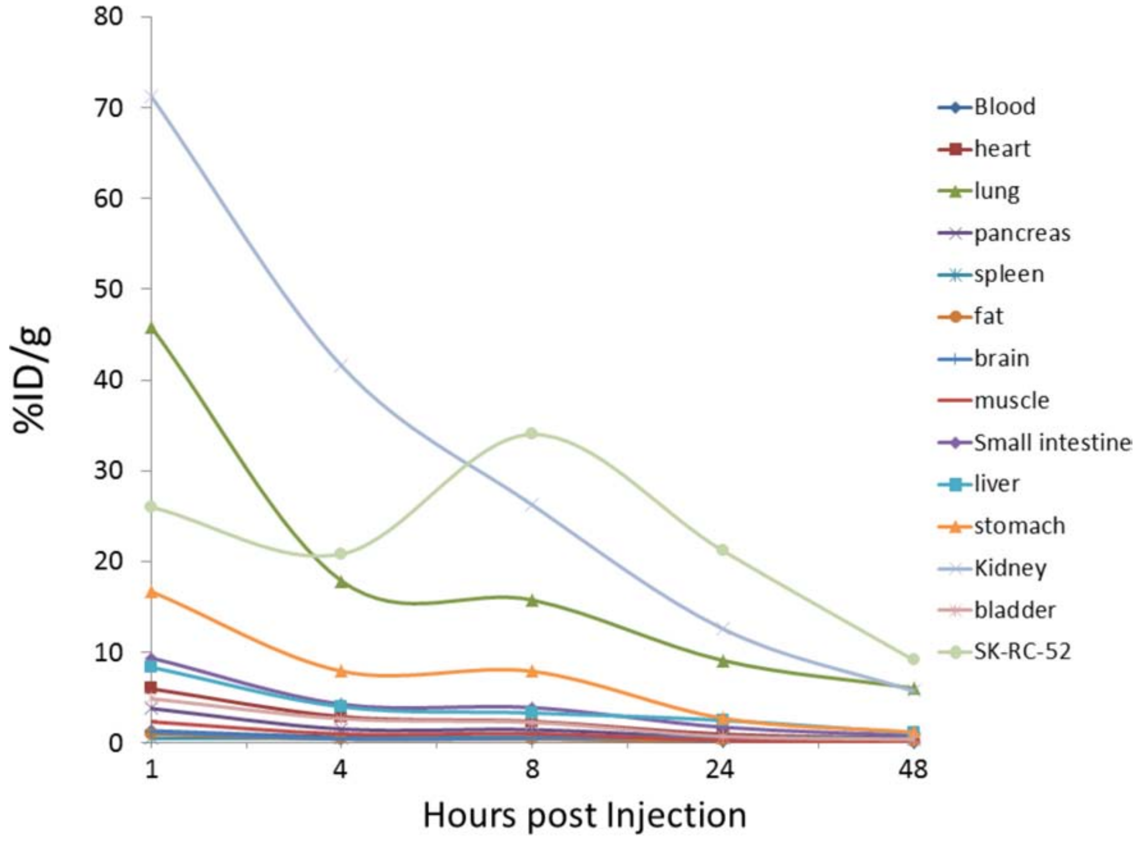

Supplementary Figure S1: Time-activity curve of [^{111}In]XYIMSR-01 for major organs and SK-RC-52 tumor.

Supplementary Table S1: Calculated half-life of [^{111}In]XYIMSR-01 for major organs and SK-RC-52 tumor

Organ	Half-life (hr)	Organ	Half-life (hr)
Blood	3.246	Muscle	2.534
Heart	3.538	Small intestine	5.597
Lung	5.602	Liver	4.243
Pancreas	3.179	Stomach	3.908
Spleen	ND	Kidney	4.344
Fat	1.536	Bladder	3.730
Brain	3.926	SK-RC-52	12.27

Biological excretion Half-life for each organ was calculated in GraphPad Prism 5 (GraphPad Software, La Jolla, CA) using the plateau followed by one phase decay exponential curve fitting with the Biodistribution data presented in the Table 1. ND: not determined.
